# Establishment and Characterization of a Stable Producer Cell Line Generation Platform for the Manufacturing of Clinical-Grade Lentiviral Vectors

**DOI:** 10.3390/biomedicines12102265

**Published:** 2024-10-04

**Authors:** Ane Arrasate, Igone Bravo, Carlos Lopez-Robles, Ane Arbelaiz-Sarasola, Maddi Ugalde, Martha Lucia Meijueiro, Miren Zuazo, Ana Valero, Soledad Banos-Mateos, Juan Carlos Ramirez, Carmen Albo, Andrés Lamsfus-Calle, Marie J. Fertin

**Affiliations:** 1 VIVEbiotech, Tandem Building, 20014 Donostia, Spain; aarrasate@vivebiotech.com (A.A.); ibravo@vivebiotech.com (I.B.); clopez@vivebiotech.com (C.L.-R.); aarbelaiz@vivebiotech.com (A.A.-S.); mugalde@vivebiotech.com (M.U.); mlmeijueiro@vivebiotech.com (M.L.M.); mzuazo@vivebiotech.com (M.Z.); avalero@vivebiotech.com (A.V.); sbanos@vivebiotech.com (S.B.-M.); albocastellanosc@gmail.com (C.A.); alamsfus@vivebiotech.com (A.L.-C.); 2 Campus of Biscay, University of the Basque Country (UPV/EHU), 48940 Leioa, Spain

**Keywords:** lentiviral vectors, gene therapy, stable producer cell line, lentiviral packaging cell line, cell line development

## Abstract

**Background/Objectives:** To date, nearly 300 lentiviral-based gene therapy clinical trials have been conducted, with eight therapies receiving regulatory approval for commercialization. These advances, along with the increased number of advanced-phase clinical trials, have prompted contract development and manufacturing organizations (CDMOs) to develop innovative strategies to address the growing demand for large-scale batches of lentiviral vectors (LVVs). Consequently, manufacturers have focused on optimizing processes under good manufacturing practices (GMPs) to improve cost-efficiency, increase process robustness, and ensure regulatory compliance. Nowadays, the LVV production process mainly relies on the transient transfection of four plasmids encoding for the lentiviral helper genes and the transgene. While this method is efficient at small scales and has also proven to be scalable, the industry is exploring alternative processes due to the high cost of GMP reagents, and the batch-to-batch variability predominantly attributed to the transfection step. **Methods:** Here, we report the development and implementation of a reliable and clinical-grade envisioned platform based on the generation of stable producer cell lines (SCLs) from an initial well-characterized lentiviral packaging cell line (PCL). **Results:** This platform enables the production of VSV-G-pseudotyped LVVs through a fully transfection-free manufacturing process. Our data demonstrate that the developed platform will facilitate successful technological transfer to large-scale LVV production for clinical application. **Conclusions:** With this simple and robust stable cell line generation strategy, we address key concerns associated with the costs and reproducibility of current manufacturing processes.

## 1. Introduction

Gene therapy has emerged as a groundbreaking alternative technology to conventional treatments for genetic and acquired diseases, seeking to modify the biological characteristics of a target cell by replacing, inactivating, or introducing genes [1]. Genetic modification can be performed in vivo when the gene therapy vector is administered directly to the patient, or ex vivo, when cells are collected from the patient or donor before gene modification and the subsequent reinfusion into the same individual (autologous transplant) or into a different patient (allogeneic transplant) [2,3]. These therapies are increasing by leaps and bounds, triggered by the promising outcomes of clinical trials over recent decades [4,5]. In this context, viral vectors have become one of the most widely used strategies for delivering genetic material [6]. Among these, lentiviral vectors (LVVs) are selected as the preferred gene therapy tool due to their high transduction efficiency, broad cellular tropism, large packaging capacity (8–10 Kb), low immunogenicity, and long-term gene expression through stable gene integration [3,7,8].

In recent years, LVVs have been employed in multiple clinical trials, with nearly 300 studies completed or ongoing [4]. Eight LVV-based gene therapies have already received regulatory approval, demonstrating both efficacy and safety [5]. Four of them are LVV-based CAR-T therapies for cancer treatment, including a therapy for acute lymphoblastic leukemia (ALL), another one for large B-cell lymphoma (LBCL), and two different treatments for relapsed or refractory multiple myeloma. Additionally, Skysona, Zytenglo, and Lyfgenia from Bluebird Bio, and Lenmeldy from Orchard Therapeutics have been approved for the treatment of active cerebral adrenoleukodystrophy (CALD), β-thalassemia, sickle cell disease (SCD), and metachromatic leukodystrophy (MLD), respectively [5,9]. Although all market-authorized LVV-therapies rely on ex vivo cell modification so far [5], efforts are underway to develop in vivo LVV-based therapies [4]. This growing demand of lentiviral vectors for emerging therapies is driving biotechnological and manufacturing companies to adapt to the market requirements.

The manufacturing of GMP-grade LVVs for clinical application typically encompasses three phases: the upstream process (USP), including cell expansion, transfection, and viral vector harvesting; the downstream process (DSP), focusing on virus purification, concentration, sterile filtration, and vector formulation before fill and finish; and lastly, the analytical characterization for final product release [3]. Focusing on the USP, the standard method for third generation LVV production consists in the transient transfection of the human embryonic kidney 293T cell line (HEK293T) with four plasmids: (a) the packaging plasmid; (b) the envelope encoding plasmid; (c) the Rev encoding plasmid; and (d) the transfer plasmid. The packaging plasmid encodes the Gag-Pol polyprotein, providing the structural elements and functional viral proteins required for viral genome retro-transcription and integration. The envelope plasmid usually encodes the heterologous VSV-G (vesicular stomatitis virus glycoprotein), which drives the vector-cell interaction and grants a broad tropism and high stability. The Rev plasmid encodes the Rev protein, which is essential for the nuclear export of the viral genome. Finally, the transfer vector plasmid transcribes the viral genome, which contains the gene of interest as well as the essential viral cis-acting elements, such as the long terminal repeats (LTR), packaging signal (ψ), or Rev responsive element (RRE), required for packaging, viral RNA transport, reverse transcription, and integration into the host cell. Furthermore, a deletion in the 3′ LTR U3 region of the transfer vector leads to self-inactivating (SIN) lentiviral vectors. This modification further secures LVV-based gene therapies by inactivating potentially packageable viral genome transcription after reverse transcription and integration [10,11,12].

This transient transfection process is easily performed at a small scale and offers flexibility to meet in a timely manner the market needs for the initial stages of product development. Despite the industry’s ability to rapidly respond to the high demand for LVVs, several aspects remain to be addressed in producing large-scale GMP-grade batches, primarily related to yields and cost-efficiency. These challenges are attributed to the limited operational control, batch-to-batch reproducibility issues, and the elevated costs of the raw material, in particular DNA and transfection reagents [9,10]. In this regard, the generation of an LVV producer cell line emerges as a promising alternative production system. An initial lentiviral packaging cell line (PCL), with the three helper genes integrated into the HEK293T genome, can reduce costs associated with helper plasmids [13,14], although transfection of the transfer vector still represents a reproducibility hurdle. For advanced clinical and commercial stages-envisioned work, stable producer cell lines (SCLs) in which the transfer vector is further integrated may represent the most cost-effective and reproducible manufacturing method since it enables a fully transfection-free LVV production.

A critical aspect of effective SCL generation for LVV manufacture is the selection of the viral envelope protein or pseudotype used for LVV packaging. While several novel strategies have been proposed to overcome the lack of permissiveness of specific target cells [15], VSV-G remains the most widely used pseudotype for clinical-grade LVV manufacture due to its broad tropism, high stability, and resistance during the manufacture process, which enables higher vector titers [15,16]. However, the generation of SCLs to produce VSV-G-pseudotyped LVVs poses a technical challenge due to the cytotoxicity associated with the constitutive expression of this envelope protein [17]. Additionally, HIV protease expressed from Gag-Pol polyprotein has also been reported to induce cytotoxic effects [10]. Thus, successful cell line generation requires the establishment of an inducible gene expression system [18,19]. The tetracycline-regulated promoter system is the predominant choice for inducible packaging and stable cell line, with two variants described [20]: the Tet-Off system, where transcription is inhibited in the presence of tetracycline (Tc) or its analogue doxycycline (DOX); and the Tet-On system, where transcription is activated in presence of Tc or DOX. To date, there are several SCLs in which the integrated helper genes are controlled by Tet-inducible systems, although mainly Tet-Off variants [21,22,23]. However, the choice of inducible technology must consider the advantages and disadvantages of each option, in particular the strength of induction and the ease of removing the inducer molecule in downstream steps.

**Figure 1:**
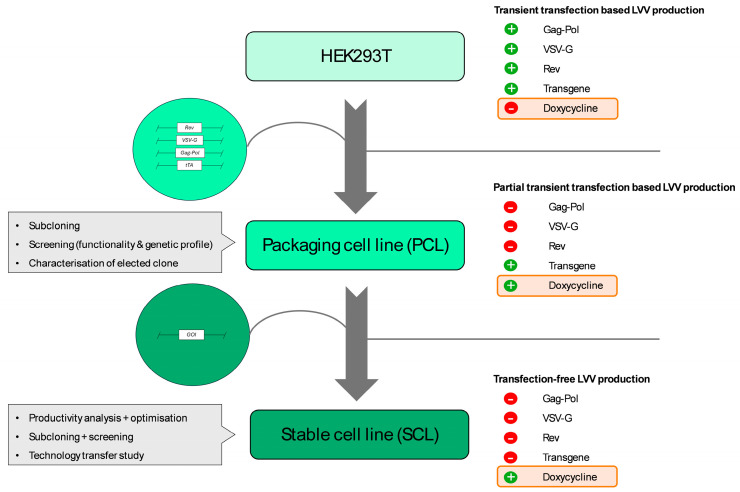
Schematic representation of the workflow followed for the development of LVV producer stable cell line generation platform.

Building on this background, this study reports the implementation of a straightforward SCL generation platform based on a well-characterized Tet-On inducible PCL (Figure 1). We demonstrate the development of an efficient transfection-free LVV manufacture based on a low dose induction with proven genomic and functional stability suitable for seamless integration into industrial upstream and downstream processes. In accordance with GMP guidelines for advanced therapy medicinal products (ATMPs) manufacturing [24], extensive analyses were performed on generated cell lines to evaluate their functionality and stability. The results of this article support that these cell lines will help to address the concerns related to large-scale GMP manufacturing, enabling more accessible gene therapy treatments to patients.

## 2. Materials and Methods

### 2.1. Plasmid Design and Cloning

Helper and transfer constructs were designed using Snapgene 5.1.7 software. DNA fragments were ordered to Integrated DNA Technologies (IDT, Newark, NJ, USA) and cloned following the In-Fusion^®^ HD cloning kit (Takara Bio Inc., Kusatsu, Japan) instructions. Prior to transfection, the bacterial elements unnecessary for LVV production and establishment in the cell line were removed through restriction enzyme digestion (FastDigest, Thermo Fisher Scientific Inc., Waltham, MA, USA) following the provider’s guidelines. The tTA plasmid was amplified by PCR by using CloneAmp HiFi PCR Premix (Takara Bio Inc.) following the provider’s recommendations. DNA was separated by 1% agarose gel electrophoresis, fragments of interest were excised and purified by NucleoSpin Gel and PCR Clean-up Kit (Macherey-Nagel GmbH & Co., KG, Düren, Germany) according to the provider’s instructions, and DNA was quantified using NanoDrop 2000 (Thermo Scientific).

### 2.2. Cell Culture and Media

HEK293T cells (ATCC CRL-3216), PCLs, and SCLs were maintained in Dubelcco’s modified Eagle’s medium with high glucose supplemented with Glutamax (DMEM high glucose from Fisher Scientific, N.H.), 10% fetal bovine serum (FBS, Merck, Darmstadt, Germany), 100 IU/mL penicillin, and 100 µg/mL streptomycin (complete DMEM). Additionally, selective pressure was maintained weekly for PCL by adding 250 µg/mL zeocin (InvivoGen, San Diego, CA, USA), 6 µg/mL blasticidin (InvivoGen), and 6.25 µg/mL hygromycin (InvivoGen) to the media. For SCL, culture was further supplemented with 0.18 µg/mL of puromycin (Sigma-Aldrich, St. Louis, MO, USA).

### 2.3. Cell Line Generation: Transfection and Selection

PCL was generated based on sequential transfection of a specific clone isolated from the HEK293T cell line (ATCC CRL-3216) with the linearized helper plasmids (Figure 1). Cells were seeded in multi-well-6 culture plates (Corning, Tewskbuy, MA, USA), and twenty hours later, when the cells were at 40–50% confluency, the specific plasmids for each case were transfected. The DNA concentration to transfect was determined by accounting for the molecular weight of each plasmid to ensure a specific molarity of each helper with respect to the others. Helper plasmids were integrated through two sequential transfections. *VSV-G*, *Gag-Pol*, and *tTA* were first co-transfected at ratio 2:1:1 (*Gag-Pol*:*VSV-G*:*tTA*). Then, *Rev* was cotransfected with additional *tTA* at the ratio of 4:1 (*Rev*:*tTA*). The transfection protocol was performed using polyethylenimine PEIpro^®^ (Polyplus, Illkirch-Graffenstaden, France) as a transfection reagent with a 2:1 PEI:DNA (*w*/*w*) ratio following supplier recommended conditions. Three days later, antibiotics were added to the media for selection at the following concentrations: zeocin (InvivoGen) 250 µg/mL, blasticidin (InvivoGen) 6 µg/mL, and hygromycin (InvivoGen) 6.25 µg/mL. In the same way, SCL was created by additional transfection of the linearized transgenes (TGs), and selection was performed with 0.18 µg/mL puromycin (Sigma-Aldrich).

### 2.4. LVV Production

For standard transient transfection based on HEK293T, cells were seeded in a multi-well-6 culture plate (Corning) and transfected twenty hours later when they reached a 70–80% confluency. Cells were transfected with the four plasmids (8.8 µg DNA/mL) along with the PEIpro (Polyplus) transfection reagent in a 2:1 PEI:DNA (*w*/*w*) ratio. Viral supernatant (VSN) was harvested 72 h after transfection and filtered using a 0.45 µm filter (Millex-HA, Merck) to later aliquot and freeze at −80 °C until further analyses. Third-generation LVVs from PCL were produced by transfection of the transfer plasmid using the same conditions as a standard transfection. Four hours after transfection, doxycycline hydrochloride (Sigma-Aldrich) was added at a concentration of 1 µg/mL.

For the third generation of LVVs produced from SCL, 18 h after cell seeding, the media was changed to complete DMEM supplemented with doxycycline hydrochloride. Productions for clone screening study and doxycycline dosage experiment (from 2 µg/mL to 1 ng/mL doxycycline) were performed in multi-well-24 plate (Corning), productions for AEX experiments in T175 (Corning), and the rest in multi-well-6 culture plates (Corning).

### 2.5. LVV Infective Titer Calculation

VSN were thawed at room temperature (RT). Afterwards, serial dilutions of the VSN were performed in DMEM supplemented with 8 µg/mL of Polybrene^®^ (Santa Cruz Biotechnology Inc., Dallas, TX, USA). The diluted VSN were mixed in a 1:1 volume ratio with HEK293T cell in the same media, following a two-hour incubation in a CO_2_ incubator. Finally, after addition of DMEM with 15% FBS for Polybrene^®^ dilution and to ensure cell viability, transduction was incubated for 72 h. For *GFP*-expressing LVVs, the transduction units were calculated by analyzing the transduced cells using the flow cytometer, CytoFLEX (Beckman Coulter, Brea, CA, USA). The viral titer was calculated using Formula (1).

(1) $$Infective\;titer\;\left(\frac{TU}{mL}\right)=\frac{\%\;GFP\times dilution\times No.\;\;of\;cells\;at\;transduction}{Total\;volume\;(mL)}$$

For non-fluorescent LVVs, the transduction units were measured, assessing the viral copy number integrated into the transduced cell genome by qPCR. Firstly, the transduced cell genome DNA was extracted according to the kit’s manufacturer instructions (QIAamp DNA Mini Kit, Qiagen, Hilden, Germany). Subsequently, quantitative PCR was performed utilizing SYBR Green Master Mix (ThermoFisher) and the QuantStudioTM 3 thermocycler (ThermoFisher). The primers used for the amplification of specifically retrotranscribed proviral DNA are shown in Table S1, allowing to determine the transducing event per cell (VCN/cells).

(2) $$Infective\;titer\;\left(\frac{VCN}{mL}\right)=\frac{\frac{VCN}{cell}\times dilution\times No.\;\;of\;cells\;at\;transduction}{Total\;volume\;(mL)}$$

### 2.6. ELISA for p24 Quantification

For viral particle quantification of the VSN produced by the different cell lines, p24 protein was quantified by an ELISA assay performed by the automatized Gyrolab xPloreTM instrument and the kit Gyrolab Bioaffy 1000 HC Toolbox (Gyros Protein Technologies, Uppsala, Sweden). Briefly, the p24 concentration of LVVs was analyzed in duplicate, performing a 2-fold serial dilution, and applied to a microfluidic disk. LVV total viral particles were calculated based on previous calculations of 2000 p24 molecules per virion [25].

### 2.7. Integration Copy Number Quantification and Targeted Locus Amplification (TLA)

An integrated helper genes copy number in the producer cell lines was identified by quantitative PCR. First, genomic DNA was extracted according to the kit’s manufacturer instructions (QIAamp DNA Mini Kit, Qiagen), and each helper gene was quantified by qPCR using a specific primer pair (Table S1) and a standard curve generated with DNA that includes the sequence to amplify. Targeted locus amplification (TLA) was performed by the company Solvias, the former Cergentis (Utrecht, The Netherlands), to identify the integration loci of each helper into the cell genome. Samples for this analysis were prepared following the indications of Solvias.

### 2.8. Viral Supernatant AEX Purification

The collected viral supernatant was first clarified through a 0.45 µm filter (Corning) and conditioned for DNA digestion with Trizma hydrochloride solution at pH 8 (Sigma-Aldrich) and MgCl_2_ (VWR, Radnor, PA, USA), according to endonuclease manufacturer instructions. The treatment with endonuclease DNARASE^®^ c-Lecta (VWR) was performed following manufacturer instructions. Following, viral supernatant was conditioned with NaCl solution for anion exchange chromatography (AEX) load for purification. AEX was performed in an ÄKTA pure™ system (Cytiva, Marlborough, MA, USA) using a Mustang Q XT Acrodisc chromatography membrane (Cytiva). Several samples from flowthrough and elution were collected along the process for recovery calculations.

### 2.9. PCL and SCL Subcloning

An automated single-cell dispenser, UP.SIGHT^TM^ (Cytena, Freiburg im Breisgau, Germany), was utilized to subclone PCL. Hundreds of single cells were dispensed and cultured in multi-well-96 culture plates (Corning). An integrated camera tracked the clones by recording pictures at the dispenser-cartridge nozzle and on the plate surface during colony formation over the days. Moreover, FDA 21 CFR Part 11-compatible C.STUDIO analysis software 1.1.3 was utilized to create a regulatory submission-ready clonality report. Later, monoclonal cell populations were grown in order to create cell banks and analytical assays. On the contrary, SCL subcloning was performed by the serial dilution method [26]. The harvested cell line was diluted in DMEM 10% FBS to reach a concentration of 0.25 cells per well of a 96-well plate (Corning) to ensure that only single cells were seeded. Following their growth by microscope visualization, cell populations derived from single cells were expanded for further analyses and cell bank generation.

### 2.10. Statistical Analysis

The results were analyzed using parametric tests, as they all followed a normal distribution. For comparison between two groups, a Student’s *t*-test was used (with Welch correction in case the standard deviations were different). For comparison between more than two groups, a one-way ANOVA test was utilized (with Welch correction in case there was not homogeneity of variances), followed by a multiple comparison test. When several groups were compared to a single control group, Dunnett’s multiple comparison test was used, while comparisons between every group were analyzed with a Tukey test.

## 3. Results

### 3.1. Generation of the Lentiviral Packaging Cell Line (PCL)

#### 3.1.1. Lentiviral Helper Plasmid Design and PCL Generation

**Figure 2:**
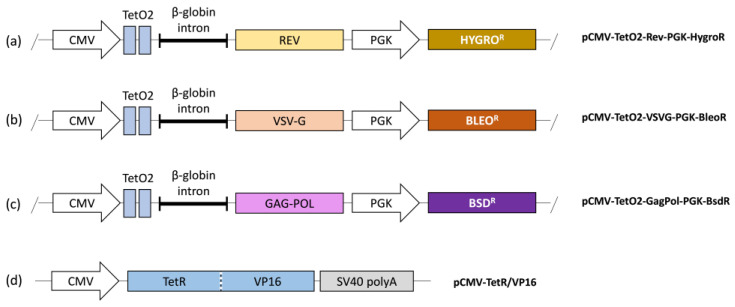
Constructs for packaging cell line generation. (**a**) Rev protein construct with hygromycin resistance gene. (**b**) VSV-G envelope construct with zeocin (bleomycin) resistance gene. (**c**) Gag-Pol polyprotein construct with blasticidin resistance gene. (**d**) Transactivator fusion protein construct.

In this study, we designed three integrable expression cassettes for the helper genes and one for the transactivator, to generate our initial PCL. The helper gene expression required for third-generation lentiviral production is controlled by an inducible promoter, CMV-TetO2, to prevent cytotoxicity (Figure 2a–c). Each helper gene construct includes a specific antibiotic resistance gene driven by a separate promoter, enabling cell selection without induction of the corresponding helper gene expression. The Tet-On transactivator fusion protein, TetR-VP16 (tTA), is expressed by a constitutive CMV promoter (Figure 2d) and does not include an antibiotic resistance gene.

The initial PCL was generated through sequential integrations of the helper genes and the transactivator into the HEK293T cell line. Each expression cassette was excised from the plasmid, purified, and integrated into the cells by transfection. Post-transfection, the modified cells were selected using the antibiotics (zeocin, blasticidin, and hygromycin) corresponding to each cassette. In addition, the integration of tTA provided a selective advantage over cells that lacked it by inhibiting the expression of the cytotoxic genes. Finally, we obtained a complete packaging cell line (PCL) after the triple-resistant cell line was stabilized and expanded.

#### 3.1.2. PCL Clone Screening Based on Functionality and Genetic Profile

**Figure 3:**
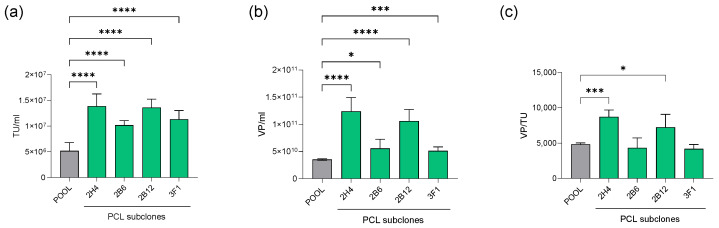
Lentiviral production capacity of the clones compared to the pool represented as (**a**) TU/mL, (**b**) VP/mL, and (**c**) VP/TU. The data were subjected to one-way ANOVA (with Welch correction for VP/mL and VP/TU) followed by Dunnett’s multiple comparison test to compare the clones to the pool. * (*p* < 0.05), *** (*p* < 0.001), **** (*p* < 0.0001).

After establishment of the PCL, cells were subcloned, resulting in more than 100 clones that underwent an initial screening based on productivity through transient transfection of a transfer plasmid (pCCL-GFP) followed by doxycycline addition (Figure S1a–c). Among all of them, four clones considerably outperformed the parental cell line (pool) productivity in harvest in terms of infectious titer in transduction units (TU/mL) and physical particles (VP/mL) measured by p24 quantification (Figure 3a,b). Data of physical and infective particles allowed the calculation of the performance factor, which assesses the number of total viral particles per infectious one (VP/TU). The performance factor for 2H4 and 2B12 showed a ratio significantly higher than the pool, indicating a lower percentage of infectious units among the total particles in harvest (Figure 3c). As opposite, the performance factor of the clones 2B6 and 3F1 remained equivalent to the pool, indicating that the infectivity of the produced particles was maintained along with the increased productivity.

**Figure 4:**
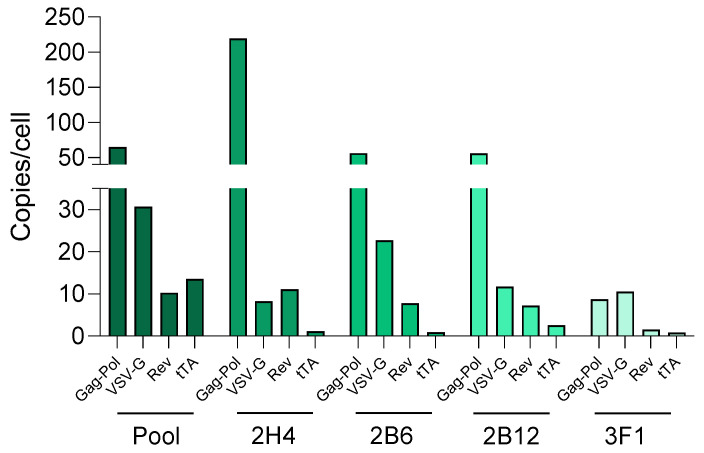
Gene integration copy number per construct and clone (copies/cell), quantified by qPCR.

For genomic characterization of the PCL clones, the integration copy number of the helper genes was quantified by qPCR (Figure 4). In general, the different cell lines displayed a similar pattern of integration: *Gag-Pol* integrations were the most represented, followed by *VSV-G*, and subsequently, *Rev* and *tTA*. However, the clone 3F1 stood out due to its lower integration number. This characteristic can be considered an advantage over the other clones since it reduces the gene disruption risks related to integrations. Moreover, 3F1 clone productivity was comparable to the others. Therefore, considering its high production capacity, the low performance factor of viral production, and its low helper integration copy number, 3F1 was selected as the optimal PCL clone for our platform. Subsequently, this clone was used for SCL generation and proof-of-concept validation within the industrial SCL platform.

#### 3.1.3. Gene Insertion Analysis of PCL 3F1 Clone

**Figure 5:**
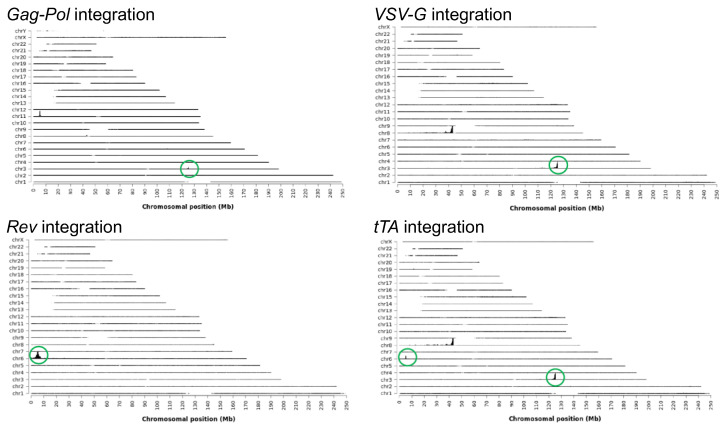
TLA sequence coverage across the human genome using specific primer sets. The chromosomes are indicated on the *y*-axis, the chromosomal position on the *x*-axis. Identified integration sites are encircled in green.

Gene integration mapping was performed by targeted locus amplification (TLA) that enables the identification of helpers-genome fusion sites by crosslinking, amplifying, and sequencing adjacent sequences. The results indicated that co-transfection of several linear plasmids led to co-integration into the same chromosomal locus, being *Rev* and *tTA* co-integrated in chromosome 6 and *Gag-Pol*, *VSV-G*, and *tTA* in chromosome 3 (Figure 5). Furthermore, data confirmed that no residual plasmid backbone was integrated, supporting the safe profile of the PCL to be used as the basis of future SCLs. Finally, two additional peaks are observed: one in chromosome 11 due to a reading artefact deriving from the homology of the vector with the genome (β-globin gene), and another peak in chromosome 8 that illustrates a complex integration event derived from a chromosomal translocation with chromosome 3.

### 3.2. Generation of Lentiviral Producer Stable Cell Lines (SCLs)

To demonstrate the effectiveness of our platform to create specific SCLs, two different proof-of-concept models were generated. First, a GFP-LVV-producing SCL was developed, as this fluorescent reporter protein, a widely used model in biotechnology, also serves as a standard model for process development. Moreover, aligning with our objective to establish a clinical-grade-envisioned SCL generation platform, we generated a clinically relevant SCL that encodes for a fusion protein, GFP-CAR. These SCLs represent a lentiviral manufacturing process based on a transfection-free method induced by doxycycline supplementation.

#### 3.2.1. Transfer Vector Design and SCL Generation

**Figure 6:**
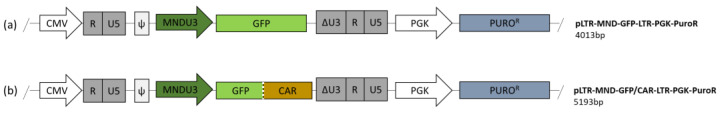
Vector genome plasmids (**a**) GFP expressing vector genome with puromycin resistance gene. (**b**) GFP-CAR fusion protein expressing vector genome with puromycin resistance gene. The length of the packaging transcript is indicated in base pairs (bp).

To generate the SCLs, two specific transgenes were designed and integrated into the genome (Figure 6a,b). In addition to the essential viral cis-acting elements, the gene of interest was expressed by the synthetic promoter MNDU3 proceeding from a modified MPSV LTR [27]. Puromycin resistance gene expression, which allows cell selection after transgene cassette integration, was controlled separately by the human phosphoglycerate kinase 1 promoter (PGK) and placed downstream of the 3′LTR to avoid its packaging into the produced LVV.

The integration of the transgenes required to generate SCL was performed through transfection into the established PCL (3F1 clone). *GFP* and *GFP-CAR* transgenes, as represented in Figure 6, were excised and purified prior to transfection. Afterwards, cells were selected under puromycin pressure to finally obtain polyclonal stable producer cell lines. The integrated transgene copy number was determined by qPCR quantification and resulted in an average of nine to ten copies integrated for the SCL-GFP pool, while the SCL-CAR pool integrated an average of two copies.

#### 3.2.2. Productivity Characterization of the SCLs

**Figure 7:**
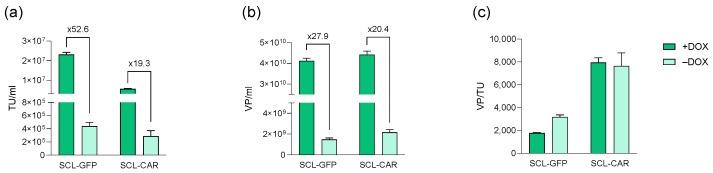
SCL-GFP and SCL-CAR production capacity in presence (+DOX) and absence (–DOX) of doxycycline as (**a**) TU/mL, (**b**) VP/mL, and (**c**) VP/TU.

LVV production of the SCLs was induced by doxycycline addition, and lentiviral production was analyzed in terms of functional and physical viral titers. Results were compared to basal LVV production yield obtained in the absence of doxycycline. In terms of biological titers, both cell lines appeared to effectively produce LVVs upon doxycycline induction (Figure 7a). SCL-GFP, with titers reaching up to 2.42 × 10^7^ TU/mL, showed a 50-fold titer increase when compared to the non-supplemented condition. In the case of SCL-CAR, the titer was increased 20-fold up to 5.9 × 10^6^ TU/mL. Physical viral particle production, assessed by p24 protein quantification, resulted in a similar production pattern for both cell lines (Figure 7b) with an approximately 20-fold increase when compared to the non-induced condition. Likewise, the performance factor of the production was calculated to evaluate the functionality of the produced LVVs (Figure 7c).

**Figure 8:**
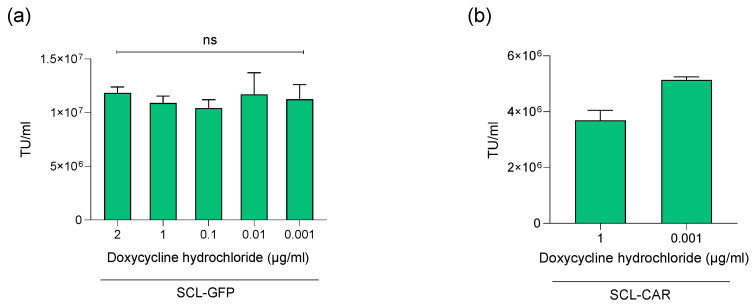
(**a**) SCL-GFP production capacity with different concentrations of doxycycline is shown in TU/mL. ANOVA one way test followed by Tukey’s multiple comparison test have been used to compare the conditions. The comparisons between the various doxycycline conditions are non-significant. (**b**) SCL-CAR production capacity with initial and lowest doxycycline concentrations shown in TU/mL.

After demonstrating the functionality of SCL generated within our platform, the process was improved by optimizing the doxycycline induction concentration (from 2 µg/mL to 1 ng/mL). All the doxycycline concentrations tested in this experiment, including the lowest one, produced a similar LVV titer (Figure 8a), demonstrating that our SCL-GFP was highly productive even with very low doxycycline concentrations. Results were further confirmed with SCL-CAR, where the lowest concentration led to a similar LVV titer compared to the previously used 1 µg/mL condition (Figure 8b).

#### 3.2.3. Subcloning of SCL-CAR and Final Clone Functionality Characterization

**Figure 9:**
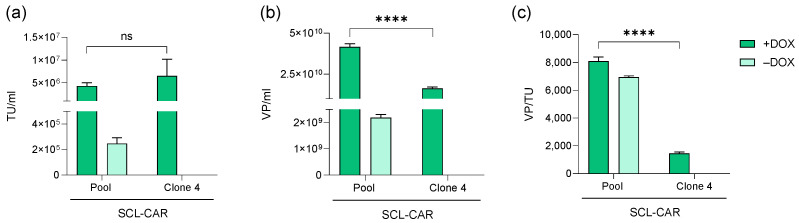
Comparison of parental SCL-CAR with SCL-CAR clone 4 in presence (+DOX) and absence (–DOX) of doxycycline in (**a**) TU/mL, (**b**) VP/mL, and (**c**) VP/TU. Data from + DOX condition were subjected to Student’s t-test (with Welch correction in VP/mL). ns = non-significant, **** (*p* > 0.0001).

Cell monoclonality is an essential recommendation of regulatory agencies to ensure consistent reproducibility across biomanufacturer batches [28]. To meet this standard, we isolated several clones and established a final SCL clone selection strategy based on productivity criteria. Accordingly, the polyclonal SCL-CAR pool was subcloned, and the resulting clones were evaluated in terms of physical and infectious titers, along with the resulting performance factor. One clone, the SCL-CAR clone 4, emerged as the top performer, producing the highest functional LVV titer among all the clones with yields similar to those obtained with its parental polyclonal population (Figure 9a). Interestingly, no detectable titers, neither physical nor infectious, were observed in the absence of doxycycline (limits of detection 1.26 × 10^6^ VP/mL and 1.5 × 10^4^ TU/mL, respectively), suggesting a complete inhibition of the helper expression in the clone 4 (Figure 9a,b). Additionally, physical viral particle quantification showed a significantly lower number of physical particles when compared to the parental pool (Figure 9b). Noteworthy, the performance factor calculated from these data shows that the performance of the LVV suspension produced by the clone 4 was 4-fold lower than the one of the parental cell line, indicating superior infectivity of the viral suspension (Figure 9c).

**Figure 10:**
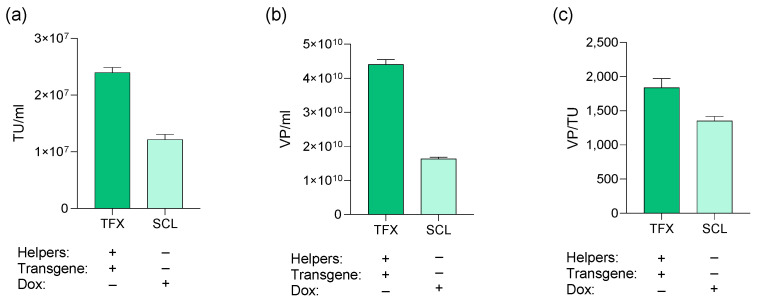
Production system comparison in (**a**) TU/mL, (**b**) VP/mL, and (**c**) VP/TU.

To compare the SCL production capacity against the transient transfection-based manufacture, the parental HEK293T cells were transfected with the same *GFP-CAR*-expressing transgene that was integrated in the SCL. Productivity was measured based on infectious and physical particles. Results showed that both systems provided LVV titers in the range of 10^7^ TU/mL. Despite SCL-CAR-based LVVs displayed a slightly lower functional titer (Figure 10a) and physical titers (Figure 10b), preliminary yields obtained by induction were comparable to our well-optimized transient transfection system. Importantly, similar performance factors for both cell lines were observed (Figure 10c), indicating comparable infectivity of production harvest.

### 3.3. Technology Transfer Suitability Analyses to Large-Scale Process

Before technology is transferred to large-scale (bioreactor) production, several crucial aspects must be addressed to achieve our goal of generating GMP-grade LVV manufacturing systems for clinical use. In particular, the cell line functionality and genomic stability must be ensured, as well as the compatibility of our industrial and well-established downstream processes with the viral harvest produced by SCL induction.

#### 3.3.1. Stability Analysis of SCL-CAR Clone 4

**Figure 11:**
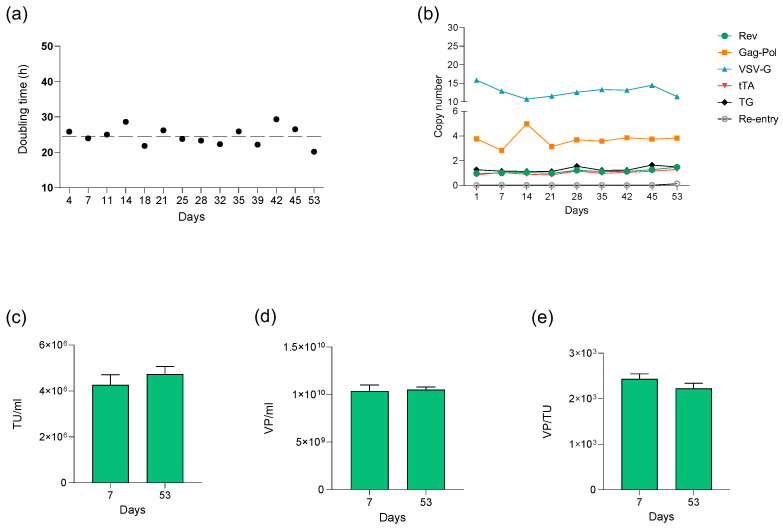
(**a**) Doubling time of the cell line in culture. Mean is shown as discontinued line. (**b**) Helper integration copy number at different days in culture. Productivity for day 7 and day 53 in culture is represented as (**c**) TU/mL, (**d**) VP/mL, and (**e**) VP/TU.

Clinical-grade LVV batches require well-controlled master and working cell banks (MCB and WCB, respectively) that enable consistent starting material along batches. Furthermore, given the needs for cell expansion for large-scale bioreactor seeding, it is fundamental to demonstrate that our SCL-CAR maintains its characteristics along cell duplications. To this end, a stability analysis was conducted across cell passages over more than 50 cell duplications to ensure the functionality and genomic stability of the SCL-CAR clone. The growth rate monitoring demonstrated a consistent doubling time with an average of 24.55 h (SD = 2.48) (Figure 11a). Genomic stability was confirmed by quantification of the helper copy number integrated in the cell line by qPCR, which demonstrated a consistent integration copy number throughout the culture period. In addition, the transduction events of the producer cell line by the newly generated LVVs, also known as retro-transduction or re-entry process, were assessed by qPCR. The basal leakage of the inducible transactivation system might indeed result in self-transduction of producer cells; however, the results indicated an absence of proviral sequence detected in the cell line (Figure 11b). Moreover, the productivity in terms of both functional and physical viral particle production was proven to be similar from the beginning to the end of the study (Figure 11c–e). Finally, basal LVV production in the absence of doxycycline was out of range of the detection limit of this quantification method (threshold 1.5 × 10^4^ TU/mL) and is therefore not represented in Figure 11.

#### 3.3.2. LVV Purification by AEX Chromatography

Given the inherent characteristic of the SCL production system in which the transfection step is eliminated, the viral supernatant generated may differ significantly from the LVV produced by traditional transfection-based methods. Thus, viral supernatant from SCL-CAR clone 4 was processed following the purification procedure established in our DSP platform up to the AEX purification step. Product recovery and contaminant removal were assessed in order to evaluate the process adjustment to the SCL-produced viral supernatant.

**Figure 12:**
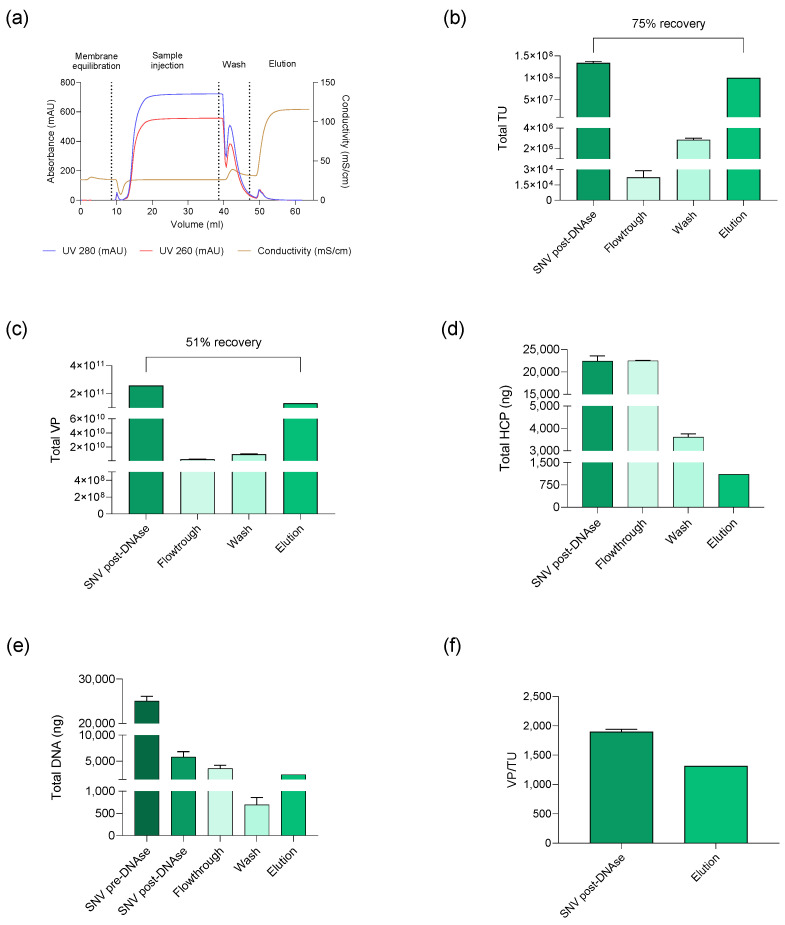
Results of AEX purification process of the supernatants produced by the SCL-CAR clone 4. (**a**) AEX chromatogram. Fractions collected in AEX procedure analyzed by (**b**) TU/mL and (**c**) VP/mL. (**d**) Total DNA in nanograms (ng). Sample DNA concentration before and after the enzymatic digestion are also displayed (pre-DNase and post-DNase). (**e**) Total HCP in nanograms (ng). (**f**) Performance factor (VP/TU) calculation of the VSN before and after AEX procedure.

The initial DSP procedure comprises three steps: VSN clarification, DNA enzymatic digestion, and AEX purification. The different fractions collected during the AEX purification (Figure 12a) were analyzed in terms of physical and infectious titer, residual host cell protein, and residual DNA. The AEX step yielded a recovery of 75% of total transduction units (Figure 12b) and 51% of physical particles produced by the SCL-CAR clone 4 (Figure 12c). Further analysis of samples from the chromatography process revealed that residual DNA in the suspension was reduced by up to 90% following AEX chromatography, aided by prior enzymatic digestion, which removed 75–80% of total DNA (Figure 12d). Host cell protein removal was nearly complete in the flowthrough, resulting in minimal protein content in the elution fraction (Figure 12e). Additionally, the performance factor determined by p24 protein quantification across fractions decreased after AEX purification, indicating an improved quality of the final product (Figure 12f).

## 4. Discussion

The continuous rise in LVV-based cell and gene therapies undergoing clinical trials has led to a subsequent increase in regulatory approvals for commercial use [4,5,9]. This steady increase signifies a challenge for biotechnological companies that need to develop reproducible, scalable, and cost-effective manufacturing processes to achieve high LVV yields with superior quality [9,10]. In large-scale LVV manufacturing, the upstream process constitutes a significant portion of the total production costs. It is estimated to account for 40% to 60% of the overall expenses depending on the specific production methods, quality grade, and scale [29,30]. One of the main contributors to this high cost is the large amount of reagents that require to be supplied at suitable quality for GMP manufacture; among them, plasmids and transfection reagents used in the traditional LVV production process [9]. Additionally, the challenges associated with the production system represent a pitfall for LVV-based gene therapies. Indeed, the stochasticity of the transfection step compromises batch-to-batch reproducibility in terms of LVV titer and quality. Transfection reagents such as the commonly used PEI depend on ionic strength, ratio to DNA, or incubation time that may result in variable complex formation. These parameters can reduce transfection efficiency and, thus, impact the manufacturing process consistency [31]. Moreover, residual plasmid DNA proceeding from the transfection step must be cleaned-up from the LVV product to avoid its transfer to patient cells along vector administration [9]. In this context, the use of an LVV producer stable cell lines emerges as an optimal alternative for an LVV production, eliminating the necessity for transfection reagents and plasmids in the LVV manufacturing process. Altogether, producer stable cell lines can reduce upstream manufacturing costs and improve lentiviral production reproducibility. Therefore, in this work, the design and development of a robust platform to generate SCLs for clinical-grade LVV manufacture is described. This platform encompasses an initial and well-characterized packaging cell line based on the third generation LVV system and the following SCL generation by further integrating the transfer vector. These SCLs permit fully transfection-free manufacture of LVVs, effectively addressing the main challenges associated with large-scale production. Furthermore, SCLs enable a significant reduction of cost of goods sold (COGS), allowing more affordable gene therapies for patients [32]. As proof of concept, we have generated two SCLs, with one undergoing further analysis, which demonstrated genomic stability along duplications (required for large-scale manufacturing processes) while maintaining consistent productivity of functional vectors.

The initial packaging cell line was generated by linear DNA transfection of the helper constructs coupled with specific antibiotic resistance genes, and modified cells were selected by adding antibiotics to the cell culture media. Along these lines, a polyclonal PCL pool was generated. Taking advantage of the inherent variability of the polyclonal cell population, a screening study of more than 100 clones was performed to select the best one in terms of LVV productivity. As expected, there was great variability between the clones, with a median value of 6.27 × 10^5^ TU/mL and an interquartile range of 2.18 × 10^6^ TU/mL. The top-performing clones were scaled up for production, ultimately leading to the selection of the four best clones. The several productivity assessments upon induction and in basal conditions, along with the genomic characterization, resulted in a monoclonal cell line that displayed superior productivity compared to the parental cell line (higher than 1 × 10^7^ TU/mL), provided by a low copy number of integrations. The genomic characterization was complemented by a TLA study, which confirmed that the co-transfection of the linear helper plasmids led to the co-integration of the constructs into two different chromosomal loci.

As proof of concept, two different SCLs were generated with distinct transfer genomes. On the one hand, *GFP*-encoding LVV producer SCL is a widely used reporter gene that allows easy production and analysis. On the other hand, a SCL with *GFP-CAR* transgene: a NKG2D receptor targeted CAR fused to a GFP to facilitate the analytical assessment. The latter was performed as an approach that emulated a therapeutic context. Productivity of the two SCLs was assessed in terms of functional and physical LVV titers, both in the presence and absence of doxycycline. The polyclonal SCLs produced up to 2.42 × 10^7^ TU/mL in the case of SCL-GFP and up to 5.9 × 10^6^ TU/mL in the case of SCL-CAR. This viral titer difference might be attributed to the larger size of the therapeutical genes as well as their biological activity which usually exerts an impact on the functional titer mainly related to reduced packaging and transduction efficiency [33,34].

With the aim to optimize the production protocol, we focused on the doxycycline concentration used to induce production. Doxycycline is needed for the activation of the Tetracycline (Tet) inducible system, which regulates the expression of the helper genes in order to mitigate the cytotoxic effects of the VSV-G and Gag-Pol-derived proteases [10,16,17]. Rev expression was also controlled by the same inducible promoter to establish a fully inducible production system. A Tet-On configuration was designed with the aim to provide a rapid LVV induction as well as feasible, large-scale manufacturing management. Doxycycline concentrations were screened to identify the optimal dose for a high LVV yield titer. The inducible system demonstrated efficient responsiveness to 1 ng/mL of doxycycline. Up to date, 2.5 ng/mL seems the lowest dose tested with similar systems according to the literature [21,35,36,37,38]. Since all the concentration conditions yielded similar productivity, the lowest concentration was selected as optimal system induction. This optimization offers the lowering of the residual doxycycline concentration in harvest, thereby alleviating DSP challenges to achieve final product quality attributes. Nevertheless, in this study, no specific quantification of doxycycline was performed; therefore, prior to accommodation of this production system for GMP-grade LVV manufacture, the implementation of an analytical method for doxycycline detection in the final product will be needed, such as a High-Performance Liquid Chromatography (HPLC) [39,40].

In agreement with regulatory recommendations related to the use of monoclonal cell line-based master and working banks for GMP manufacturing [28], we included in our platform a clonal isolation step. The obtained monoclonal cell lines were evaluated through a productivity-based screening process, both in presence and absence of induction. Overall, the best clone identified not only outperformed the titer of the parental cell line, producing titers up to 1.27 × 10^7^ TU/mL in bulk harvest, but also exhibited fully repressed LVV production in absence of inducer. Moreover, the productivity of this clone was compared to the transfection-based LVV manufacture method. Both approaches achieved high LVV titer yields, with SCL-CAR reaching 1.28 × 10^7^ TU/mL in harvest; that compares well with titers obtained by transient transfection with the same construct, which yields average titers of 2.46 × 10^7^ TU/mL.

The regulatory guidelines ICH Q5D also state that the producer cell line must retain a consistent LVV-production capacity as well as stability during and cultivation [28]. Therefore, the transferability of the SCL to large-scale manufacturing was assessed through genomic and functional stability analyses of the monoclonal stable cell line. Thus, we aged cells during an estimated duplication number required for GMP-grade LVV manufacturing, covering cell expansion for banking and production processes. During this stability study, the cell line exhibited a stable growth rate with constant integration copy numbers. Additionally, no retro-transduction events were observed, aligning with the lack of leakage production in the absence of an inducer. Taken together, genomic stability and absence of re-entry might have granted a productive consistency since the production levels of both infectious and physical particles remained similar at the beginning and the end of the culture. Despite the basal leakage expression of the helper genes in the absence of an inducer is a common feature for regulatable systems that may lead to uncontrollable LVV production [41] and a potential retro-transduction [42], our monoclonal cell line showed a potent repression of the inducible system with neither process occurring.

Once established the compliance of the SCL-CAR for upstream processing, the next step was to evaluate its suitability for downstream processing. The DSP of an LVV production intends to purify the LVV bulk harvest by removing the contaminants present in the viral suspension. One of the main steps of a typical DSP platform for LVV manufacture is the anionic exchange (AEX) chromatography. This method is based on the negatively charged LVV particles binding into the positively charged chromatography matrix [43,44]. Since our standard downstream process comprises an AEX step specifically developed in the frame of transient transfection-based manufacturing processes, we evaluated its efficiency for SCL-produced LVVs. Indeed, the transfection reagent PEI transiently alters the local charge environment during transfection due to its cationic nature [45,46]. Thus, its removal in a SCL-based production is likely to alter the particles charge and their interaction with the AEX membrane. After the purification process, most of the host cell proteins, as well as the residual DNA previously treated with endonuclease, were removed in the flowthrough. Additionally, 75% of the infectious virus were recovered in the elution fraction. These results are higher than those reported in the literature, where the recoveries oscillate around 30–60% at a large scale [47,48,49,50], although some have reached 80% recovery after optimization [51]. Also, 51% of loaded p24 was recovered in the eluted fractions, while 5% was lost in the flowthrough or wash. The remnant 44% is probably adsorbed in the membrane due to the unspecific binding of the particles to the column [52,53]. Overall, accounting for the p24 concentration and infectious titer in the elution fraction, the performance factor happens to slightly reduce in the final product after AEX chromatography, which results in an improvement in vector quality along this purification step. This preliminary evaluation of DSP performance on the SCL-produced LVV is highly promising, considering the high infectious recovery along with the high removal rate of the contaminants. However, it remains subject to further improvement through a complete DSP process development as described by Moreira et al. [54].

In conclusion, we have generated two LVV-producing stable cell lines, SCL-GFP and SCL-CAR, from a well-characterized PCL that have proved the robustness of this platform for clinically relevant SCL generation. Successful results were obtained with both SCL regarding infectious titer and physical-to-infectious particle ratio. A clonal isolation and selection upon productivity criteria was demonstrated to be beneficial in terms of viral titers. The main purification step was successfully evaluated, with the derived LVVs indicating no major hurdles in downstream process development. Results indicate that we have considerably improved the cost-effectiveness of the lentiviral manufacturing upstream process, eliminating costs associated with plasmids and transfection reagents together with an overall increase in the quality of the produced LVVs. These results pave the way for future industrialization, derisking large-scale production development. SCL-derived LVV manufacturing at the bioreactor scale will encompass the evaluation of several process parameters, such as, harvest collection timing, seeding cell density, or contaminant evaluation. Moreover, accessory analytical studies, such as HPLC assays for residual doxycycline detection or RCL studies, need to be implemented to further confirm the absence of related safety issues.

## Data Availability

The original contributions presented in the study are included in the article. Further inquiries can be directed to the corresponding authors.

## Supplementary Materials

The following supporting information can be downloaded at: https://www.mdpi.com/article/10.3390/biomedicines12102265/s1, Figure S1: Screening of monoclonal PCL based on productivity (TU/mL). (a) First production round. (b) Second production round. (c) Third production round.; Table S1: List of primers used for integration copy number quantification and infectious titer quantification through viral copy number measurement.
