# Supplementary figures and images for: Establishment and Characterization of a Stable Producer Cell Line Generation Platform for the Manufacturing of Clinical-Grade Lentiviral Vectors
Source: Biomedicines. 2024 Oct 4;12(10):2265. doi: 10.3390/biomedicines12102265 (PMC11504443; doi:10.3390/biomedicines12102265)

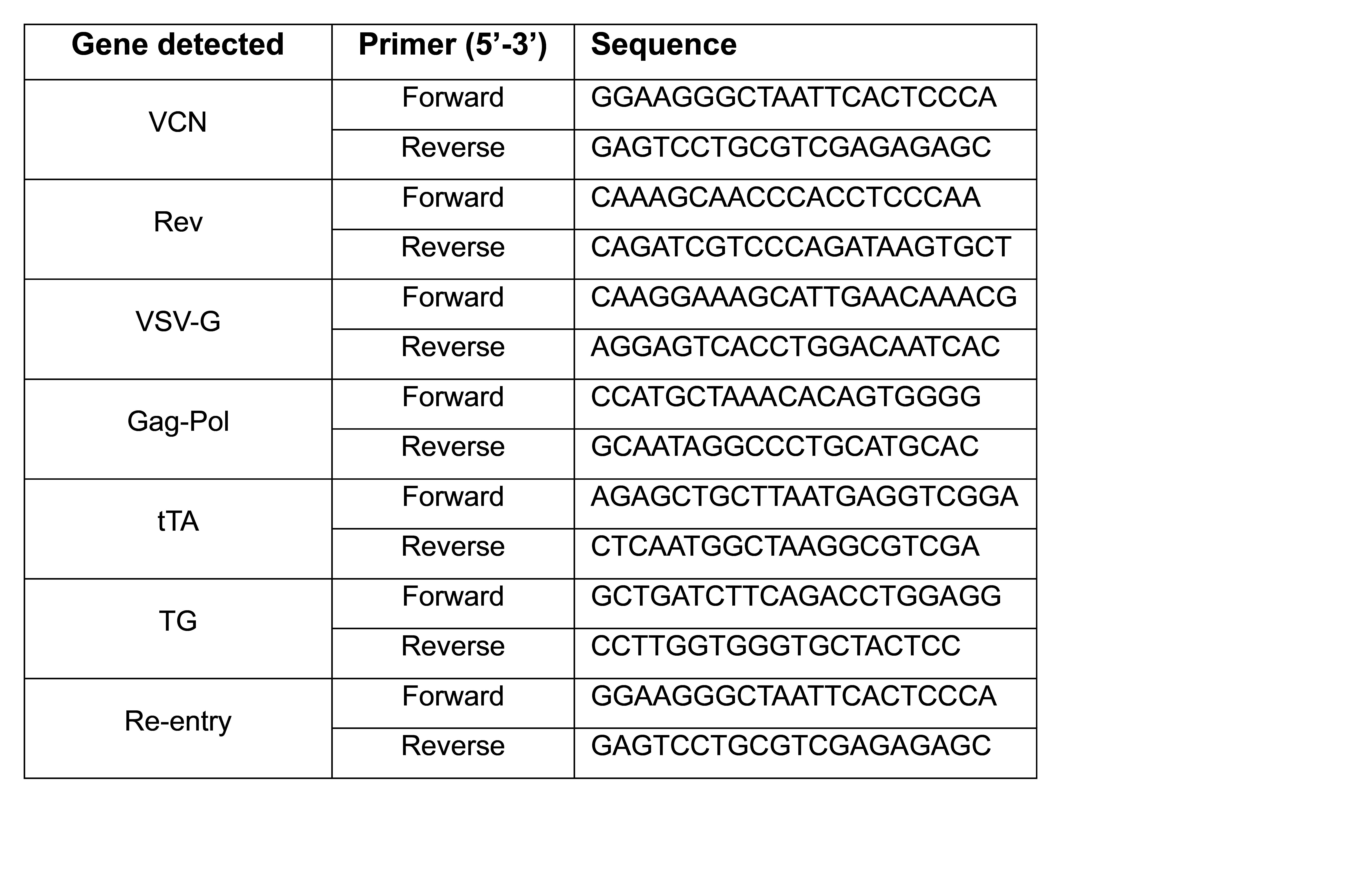

Supplement: Supplementary file 1 [file biomedicines-12-02265-s001.zip › Supplementary figures/Table S1.tif]
